# Tumor Microenvironmental Changes Induced by the Sulfamate Carbonic Anhydrase IX Inhibitor S4 in a Laryngeal Tumor Model

**DOI:** 10.1371/journal.pone.0108068

**Published:** 2014-09-16

**Authors:** Tineke W. H. Meijer, Johan Bussink, Miriam Zatovicova, Paul N. Span, Jasper Lok, Claudiu T. Supuran, Johannes H. A. M. Kaanders

**Affiliations:** 1 Department of Radiation Oncology, 874 Radboud university medical center, HB Nijmegen, The Netherlands; 2 Department of Molecular Medicine, Institute of Virology, Slovak Academy of Sciences, Bratislava, Slovak Republic; 3 Laboratorio di Chimica Bioinorganica, Polo Scientifico, Università degli Studi di Firenze, Sesto Fiorentino, Florence, Italy; National Health Research Institutes, Taiwan

## Abstract

**Background and Purpose:**

Carbonic anhydrase IX (CAIX) plays a pivotal role in pH homeostasis, which is essential for tumor cell survival. We examined the effect of the CAIX inhibitor 4-(3′(3″,5″-dimethylphenyl)-ureido)phenyl sulfamate (S4) on the tumor microenvironment in a laryngeal tumor model by analyzing proliferation, apoptosis, necrosis, hypoxia, metabolism and CAIX ectodomain shedding.

**Methods:**

SCCNij202 tumor bearing-mice were treated with S4 for 1, 3 or 5 days. CAIX ectodomain shedding was measured in the serum after therapy. Effects on tumor cell proliferation, apoptosis, necrosis, hypoxia (pimonidazole) and CAIX were investigated with quantitative immunohistochemistry. Metabolic transporters and enzymes were quantified with qPCR.

**Results:**

CAIX ectodomain shedding decreased after treatment with S4 (p<0.01). S4 therapy did neither influence tumor cell proliferation nor the amount of apoptosis and necrosis. Hypoxia (pimonidazole) and CAIX expression were also not affected by S4. CHOP and MMP9 mRNA as a reference of intracellular pH did not change upon treatment with S4. Compensatory mechanisms of pH homeostasis at the mRNA level were not observed.

**Conclusion:**

As the clinical and biological meaning of the decrease in CAIX ectodomain shedding after S4 therapy is not clear, studies are required to elucidate whether the CAIX ectodomain has a paracrine or autocrine signaling function in cancer biology. S4 did not influence the amount of proliferation, apoptosis, necrosis and hypoxia. Therefore, it is unlikely that S4 can be used as single agent to influence tumor cell kill and proliferation, and to target primary tumor growth.

## Introduction

Tumor metabolism produces large amounts of acids by converting glucose into lactate acid and protons through glycolysis, and carbon dioxide (CO_2_) through oxidative phosphorylation and the pentose phosphate pathway [1]. Since many intracellular processes, such as ATP production, protein synthesis and cell proliferation, require a close regulation of the intracellular pH, tumor cells must develop strategies to protect the cytosol from cytotoxic acidosis and to survive [2]. An important regulator of pH homeostasis is the hypoxia-inducible factor 1 (HIF-1), which enhances the expression of several membrane-located transporters and enzymes including monocarboxylate transporters (MCT), and carbonic anhydrase IX and XII (CAIX, CAXII) [1]–[3]. Also sodium-hydrogen exchangers (NHE) and plasma membrane proton pump vacuolar ATPase (V-ATPase) are involved in pH regulation [3]. Overexpression of the V-ATPase ATP6V1C1 mediates intracellular pH regulation in head and neck squamous cell carcinomas [4]. Tumor pH homeostasis maintains a slightly alkaline intracellular pH (7.2–7.4), whereas the extracellular pH is more acidic (6.5–7.0) [3], [5].

CAIX and CAXII are transmembrane zinc-containing metalloenzymes that catalyze the reversible hydration of carbon dioxide into bicarbonate and protons. Since the active site of CAIX and CAXII resides in the extracellular space, this enzymatic reaction contributes to extracellular acidification, which promotes tumor cell migration, invasion and metastasis formation [2], [3], [6]. Furthermore, CAIX and CAXII are involved in maintaining an alkaline intracellular pH, as the bicarbonate ion resulting from the catalytic reaction can be imported into the cell through chloride/bicarbonate exchangers and sodium/bicarbonate co-transporters. This intracellular alkalinization supports cell growth and survival [2], [3], [5]–[7]. CAIX has higher extracellular activity than CAXII [7]–[9]. In normal tissues, CAIX is only expressed in the mucosa of the glandular stomach, large bile ducts and peritoneal lining, while CAXII is also expressed in the urinary tract, and skin and soft tissues. CAIX overexpression is found in several types of solid tumors including head and neck cancer, lung carcinomas, esophageal cancer and soft tissue sarcomas. In most studies, CAIX expression is associated with a poor prognosis [2], [3], [10], while the prognostic significance of CAXII is controversial [2], [11], [12].

On the basis of the prognostic significance of CAIX and its important role in pH regulation, this enzyme could be a pivotal target for cancer therapy. Therefore, several CA inhibitors have been developed including the sulfonamides and their isoesters (sulfamates, sulfamides) [13]. These targeting agents inhibit CAIX and CAXII by binding to the catalytic zinc ion in the active site of the enzyme and thereby blocking its function [3]. Of this group of inhibitors, 4-(3′(3″,5″-dimethylphenyl)-ureido)phenyl sulfamate (S4) seems to be a very potent one, because of the high CAIX and CAXII affinity with weak inhibitory capacity towards CAI, which is highly abundant in red blood cells [14].

S4 is highly effective as an antiproliferative agent through disruption of pH homeostasis and thereby intracellular processes in 6 different breast cancer cell lines and in colorectal cancer cell lines [14], [15]. The aim of this study was to examine the effect of treatment with S4 on the tumor microenvironment in terms of the anti-proliferative capacity of S4, pro-apoptotic and pro-necrotic efficacy, and S4-induced changes in hypoxia, metabolism and CAIX ectodomain shedding. A laryngeal carcinoma tumor model was chosen based on the association of CAIX expression with a poor prognosis in head and neck cancer [2].

## Materials and Methods

### Ethics statement

The experiment was approved by the Animal Experiments Committee of the Radboud university medical center (Permit Number RU-DEC 2012-010).

### Xenograft tumor model and treatment

In this experiment, the tumor model SCCNij202 was used, which is derived from a human moderately differentiated T4N0M0 transglottic laryngeal squamous cell carcinoma.

A power analysis was performed to determine the number of animals needed to reach statistical significance using the formula n = 1+2C(s/d)^2^, where s is the standard deviation, d is the difference to be detected and C is a constant dependent on the value of α and β. Using an α of 0.05 and a 1-β of 0.8 results in a C-value of 7.85. To be able to detect an absolute difference of 7.5% between groups with an assumed standard deviation of 4.5%, then n = 1+15.7(0.045/0.075)^2^ = 6.65 = 7 mice per group.

Viable 1 mm^3^ SCCNij202 tumor pieces were implanted subcutaneously on the hind leg of 6–10 week-old athymic BALB/c nu/nu female mice. Implantation of tumor pieces was performed under isoflurane anesthesia. Animals were kept in a specific pathogen-free unit according to institutional guidelines. The mice were housed in sterile cages (maximum 11 mice per cage) with bedding and water, and food provided ad libitum. No adverse events occurred and weight of the animals was stable during the experiment. S4 was developed by the research group of C. Supuran (University of Florence, Italy) [15].

The experiment started when tumors reached a mean diameter of 6–8 mm. The experimental set-up existed of one control group and 4 treatment groups, 7 mice per group. Mice were randomly assigned to the control or treatment groups. Animals were injected with 0.25 ml vehicle i.p. (37.5% poly ethylene glycol (PEG) 400/12.5% ethanol/50% 0.9% NaCl) a day for 5 consecutive days (control group), or with 125 mg per kg body weight S4 dissolved in 37.5% PEG400/12.5% ethanol/50% 0.9% NaCl a day for 1, 3 or 5 consecutive days. Tumors were harvested 8 hours after the last i.p. injection. Two treatment groups received one S4 i.p. injection. Tumors of these groups were harvested 8 hours and 24 hours after the last i.p. injection.

To determine the effects of S4 on proliferation and hypoxia, animals were injected with 80 mg/kg of the hypoxia marker pimonidazole (Natural Pharmaceuticals International Inc., Research Triangle Park, NC, USA) one hour before euthanization and with 50 mg/kg of the proliferation marker bromodeoxyuridine (BrdUrd) (Sigma, St Louis, MO, USA) 15 minutes before euthanization. Pimonidazole i.p. injection was not successful in one case. Mice were sacrificed by means of cervical dislocation. Tumors were snap frozen in liquid nitrogen immediately after harvest.

### Immunohistochemistry

Consecutive sections of 5 µm were stained for CAIX/pimonidazole/blood vessels, CAIX/BrdUrd/blood vessels/cell nuclei, and CAIX/caspase-3/blood vessels.

Consecutive steps of the staining procedure were performed as described previously [16]–[18]. The antibody against pimonidazole was a gift of J.A. Raleigh (University of North Carolina). The antibodies against CAIX, BrdUrd and caspase-3 were purchased from Novus Biologicals (Littleton, Colorado, USA), Genetex (Irvine, California, USA) and R&D Systems (Minneapolis, Minnesota, USA) respectively. 9F1, a rat monoclonal antibody against mouse endothelium, was a gift of the Department of Pathology, Radboud university medical center. Primary antibodies were detected by appropriate Cy3-conjugated (Jackson Immuno Research Laboratories Inc., West Grove, PA, USA), Alexa488-conjugated or Alexa647-conjugated (Molecular Probes, Leiden, The Netherlands) secondary antibodies. Cell nuclei were stained with Hoechst 33342 (Sigma, Zwijndrecht, the Netherlands).

### Image acquisition and analysis

Researchers (T.M. and J.L.) were blinded for the treatment arms during the analysis of the stained sections. Slides were scanned using a high-resolution 12-bit CCD camera (Coolsnap HQ, Roper Scientific Inc., Trenton, NJ, USA) on a fluorescence microscope (Axioskop, Zeiss, Göttingen, Germany). For each staining, the exposure time was manually set before scanning. Thereafter, each section was automatically and completely scanned at 100× magnification (on average 144 fields) to yield grey scale images of the different fluorescent signals. To convert the grey scale images into binary images, thresholds for the fluorescent signals were interactively set at intensities where the steepest gradient occurred between background and foreground intensity levels [19], [20]. The tumor area was marked on the grey scale images, which excluded non-tumor tissue, necrosis, keratinization and staining artifacts. To allow optimal discrimination between tumor and non-tumor tissue, a consecutive section was stained by haematoxylin and eosin (H&E). Using ImageJ software (NIH, Bethesda, MD, USA), fractions of pimonidazole, CAIX and caspase-3 were calculated by dividing the tumor area positive for the marker by the total tumor area [19], [20]. The BrdUrd labeling index (LI) was calculated by dividing the nuclear area positive for BrdUrd by the total nuclear area of the tumor [20]. To determine the amount of hypoxia, proliferation and apoptosis within CAIX positive areas, i.e. the tumor regions in which S4 should be theoretically effective, a binary closing operation on the CAIX and pimonidazole signal was performed enabling the assessment of the colocalization of the membrane-bound CAIX with the cytoplasmic stainings (caspase-3, pimonidazole) or nuclear staining (BrdUrd). To measure the necrotic fraction, the necrotic area was marked on the grey scale images. Necrotic fraction was calculated by dividing the necrotic area by the total tumor area (including the necrotic area).

### Quantitative polymerase chain reactions (qPCR)

Total RNA from fresh frozen tumors (5 sections of 20 µm) was isolated with the Norgen total RNA purification kit (Norgen Biotek Corp., Thorold, Canada) and reversed transcribed using the iScript cDNA synthesis kit (Bio-Rad Laboratories Inc., Richmond, California, USA) with 1 µg RNA as input. mRNA levels of the metabolic markers glucose transporter 1 (GLUT1), CAIX, MCT1, MCT4, NHE1 and V-ATPase ATP6V1C1 were assessed using qPCR. The C/EBP homologous protein (CHOP), of which the mRNA level increases with decreasing pH, induces growth arrest and apoptosis after endoplasmatic reticulum stress or DNA damage. mRNA level of matrix metallopeptidase 9 (MMP9), which is involved in breakdown of the extracellular matrix, declines with decreasing pH [21]. qPCR was performed with specific primers for CHOP (FW: 5′-GGAGCATCAGTCCCCCACTT-3′, RV: 5′-TGTGGGATTGAGGGTCACATC-3′), GLUT1 (FW: 5′-GAGCATCATCTTCATCCC-3′, RV: 5′-TCTTTAGCACACTCTTGG-3′), CAIX (FW: 5′-GAGGCCTGGCCGTGTTG-3′), RV: 5′-CTGAGCCTTCCTCAGCGATT-3′), MCT1 (FW: 5′-TCTGTGTCTATGCGGGATTCTT-3′, RV: 5′-TTGAGCCGACCTAAAAGTGGT-3′), MCT4 (FW: 5′-GTTGGGTTTGGCACTCAACT-3′, RV: 5′-GAAGACAGGGCTACCTGC-3′), NHE1 (FW: 5′-ACCACGAGAACGCTCGATTG-3′, RV: 5′-ACGTGTGTGTAGTCGATGCC-3′), and the V-ATPase ATP6V1C1 (FW: 5′-GAGTTCTGGCTTATATCTGCTCC-3′, RV: 5′- GTGCCAACCTTTAAGTCAGGAAT-3′) on a CFX96 real-time PCR detection system (Bio-Rad Laboratories Inc, Richmond, California, USA) using SYBR Green. MMP9 qPCR was performed in Taqman Universal Master Mix with the FW primer 5′-ACGCACGACGTCTTCCAGTAC-3′ and the RV primer 5′-TAGGTCACGTAGCCCACTTGGT-3′, and with the Taqman probe 5′-TCCGGGAACTCACGCGCCAG-3′ for signal detection. Levels are expressed as ratios of hypoxanthine-guanine phosphoribosyl transferase (HPRT). CAIX qPCR analysis was not successful in one case.

### ELISA CAIX ectodomain shedding

Blood was collected through cardiac puncture immediately after euthanization. Blood was centrifuged at 3000 rpm for 10 minutes using the Eppendorf Centrifuge 5804 R and the resulting serum was stored at −80°C until further processing.

ELISA, which specifically recognizes the human CAIX ectodomain, was performed as described previously [22], [23]. The capture antibody V/10 (10 µg ml^−1^) was immobilized on the surface of microplate wells overnight at 4°C. After blocking and washing, the serum samples diluted 1∶2 in PBS were added to the coated wells for an overnight binding at 4°C. The attached antigen was then allowed to react with biotinylated MAb M75 diluted 1∶5000 (200 ng ml^−1^) in PBS. The amount of bound detector antibody was determined after 1 hour incubation with the peroxidase-conjugated streptavidin (Pierce) using a peroxidase substrate orthophenylene diamine (Sigma).

### Statistics

Statistical analyses were performed using Prism 4.0c (GraphPad Software, Inc., LA Jolla, CA, USA). Changes in fractions, BrdUrd LI and HPRT ratios were tested for significance using the Kruskal-Wallis test with Dunn's post test. Two-sided p-values <0.05 were considered statistically significant.

## Results

### CAIX expression in the SCCNij202 laryngeal carcinoma xenograft model

**Figure 1:**
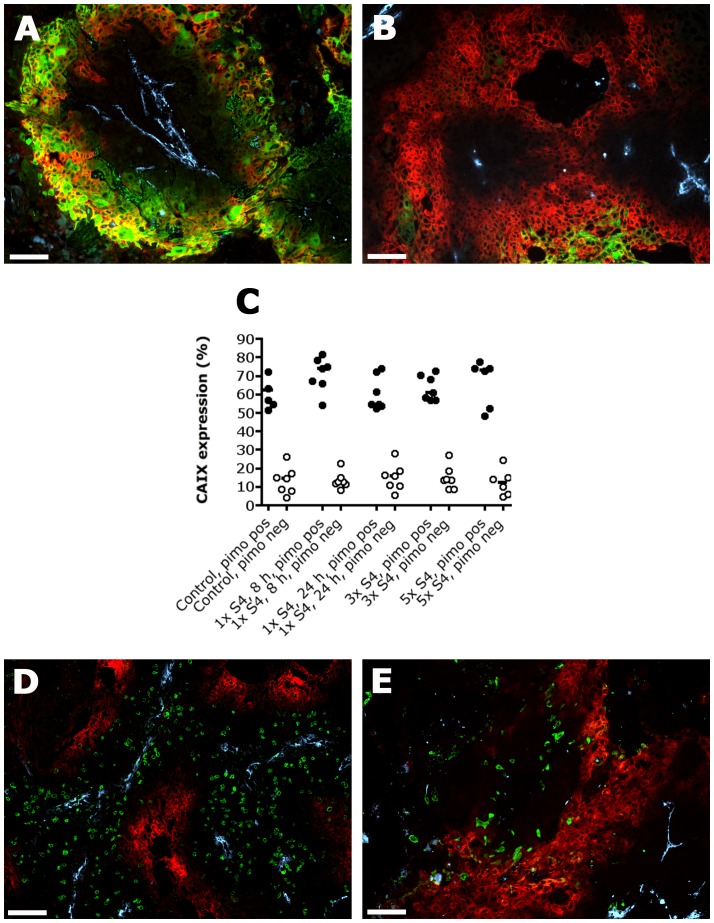
Immunofluorescent images showing CAIX expression, pimonidazole, apoptosis and proliferation in SCCNij202. CAIX is expressed in hypoxic regions as assessed by pimonidazole staining (A), but is also observed in non-pimonidazole areas (B). Yet, most of CAIX is expressed in pimonidazole positive regions (C). Proliferation (BrdUrd) and apoptosis (caspase-3) in relation to CAIX expression are shown in figure D and E respectively. *Red*, CAIX; *Green*, pimonidazole (A–B), BrdUrd (D) or caspase-3 (E); *Yellow*, overlap of CAIX (red) and pimonidazole (green); *Light blue*, vessels. Magnification 100×. Scale bars represent 100 µm. Closed circles represent CAIX expression in pimonidazole positive regions; open circles represent CAIX expression in pimonidazole negative regions. Abbrevations: 1× S4, 8 h, one i.p. injection S4, harvest after 8 hours; 1× S4, 24 h, one i.p. injection S4, harvest after 24 hours; 3× S4, one i.p. injection S4 a day for 3 days, harvest 8 hours after the last injection; 5× S4, one i.p. injection S4 a day for 5 days, harvest 8 hours after the last injection.

The SCCNij202 laryngeal xenograft model was selected on the basis of its high CAIX expression, thereby expecting a large therapeutic efficacy of S4. CAIX was often expressed in hypoxic regions as assessed by pimonidazole staining (Figure 1A). However, CAIX expression was also observed in non-pimonidazole areas, which could indicate intermediate hypoxia as CAIX is upregulated below a pO_2_ of 20 mmHg and pimonidazole below a pO_2_ of 10 mmHg (Figure 1B) [24]. Furthermore, other tumor microenvironmental conditions like acidification, tumor suppressors and oncogenic signaling pathways are able to increase CAIX expression [25]. Yet, most of CAIX was expressed in pimonidazole positive regions in both the control and the treatment groups (Figure 1C). Proliferative and apoptotic tumor cells were predominantly found in CAIX negative regions (Figure 1D–E).

### Effect of CAIX inhibition on the tumor microenvironment in a laryngeal tumor model

**Figure 2:**
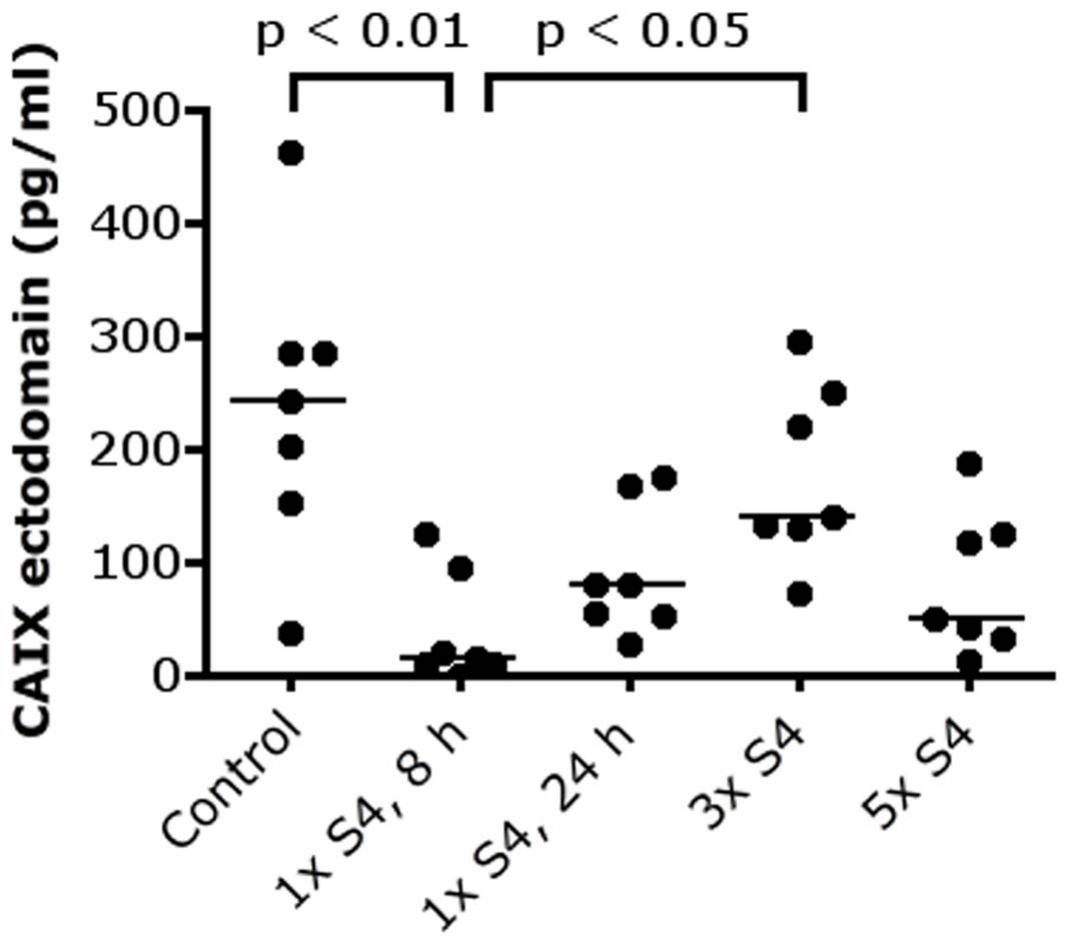
CAIX ectodomain shedding after treatment with S4. S4 decreases CAIX ectodomain shedding in the serum, especially 8 hours after the first injection. Abbreviations: CAIX, carbonic anhydrase IX; 1× S4, 8 h, one i.p. injection S4, harvest after 8 hours; 1× S4, 24 h, one i.p. injection S4, harvest after 24 hours; 3× S4, one i.p. injection S4 a day for 3 days, harvest 8 hours after the last injection; 5× S4, one i.p. injection S4 a day for 5 days, harvest 8 hours after the last injection.

CAIX inhibition using S4 did significantly decrease the amount of CAIX ectodomain shedding in the serum, especially eight hours after the first S4 injection (p<0.01) (Figure 2). Median value of the CAIX ectodomain in the serum declined from 242 pg/ml (control group) to 13 pg/ml 8 hours after one S4 injection. After 3 S4 injections, median CAIX ectodomain shedding increased to 139 pg/ml (p<0.05) (Figure 2).

**Figure 3:**
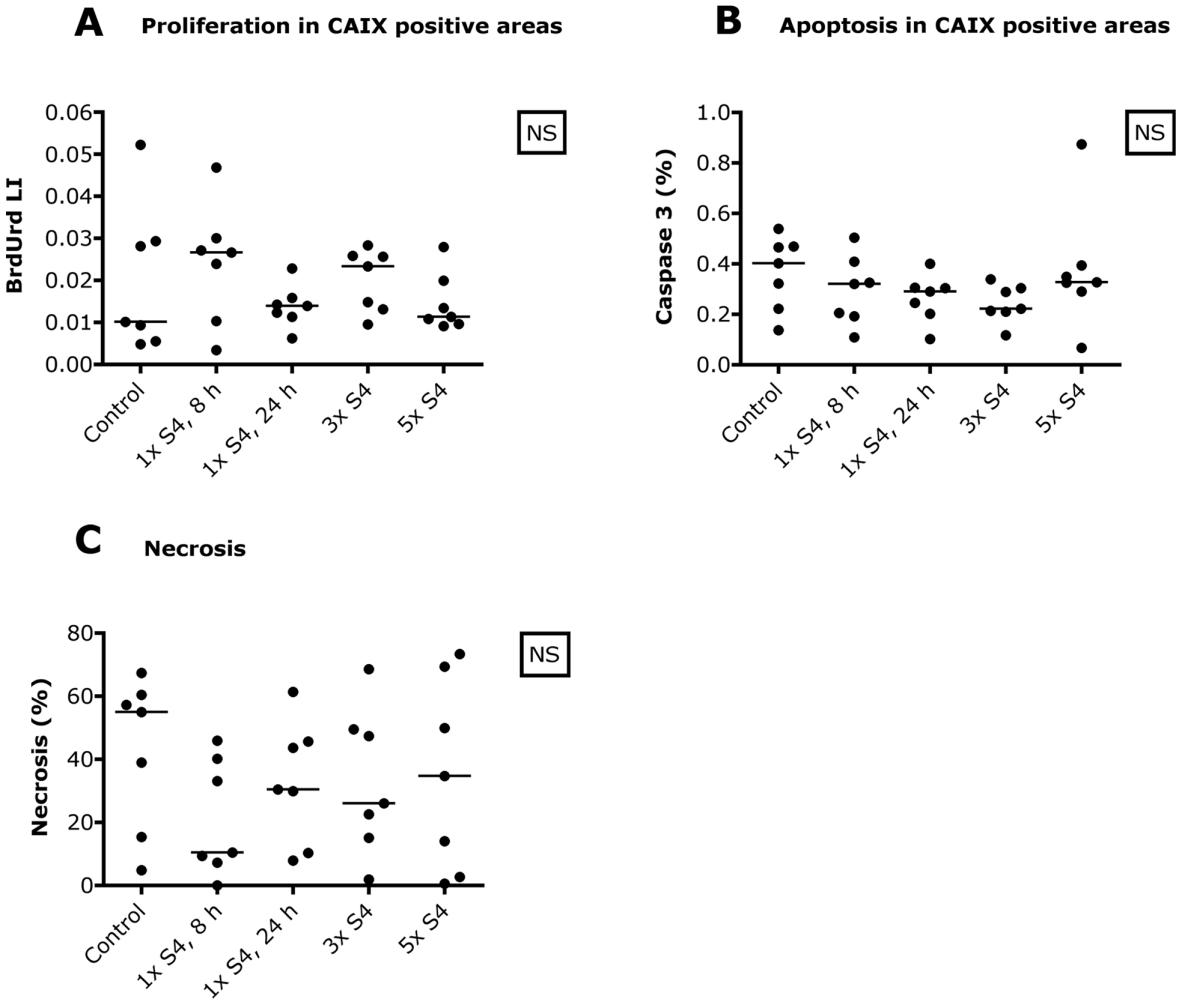
No cytotoxic effects of CAIX inhibition in SCCNij202. S4 does not affect proliferation within CAIX positive areas (A), apoptosis within CAIX positive areas (B) or the amount of necrosis (C). Abbreviations: BrdUrd, bromodeoxyuridine; CAIX, carbonic anhydrase IX; LI, labeling index; NS, not significant, 1× S4, 8 h, one i.p. injection S4, harvest after 8 hours; 1× S4, 24 h, one i.p. injection S4, harvest after 24 hours; 3× S4, one i.p. injection S4 a day for 3 days, harvest 8 hours after the last injection; 5× S4, one i.p. injection S4 a day for 5 days, harvest 8 hours after the last injection.

Median expression of CAIX and pimonidazole in the control group was 23.5% (range 16–30%) and 28.5% (range 12–39%) respectively. S4 changed neither CAIX protein expression nor the hypoxic fraction. Treatment with S4 did not influence the amount of cell proliferation and apoptosis, neither within the total tumor area nor within CAIX expressing regions (Figure 3A–B). Also the necrotic fraction was not affected by treatment with S4 (Figure 3C).

**Figure 4:**
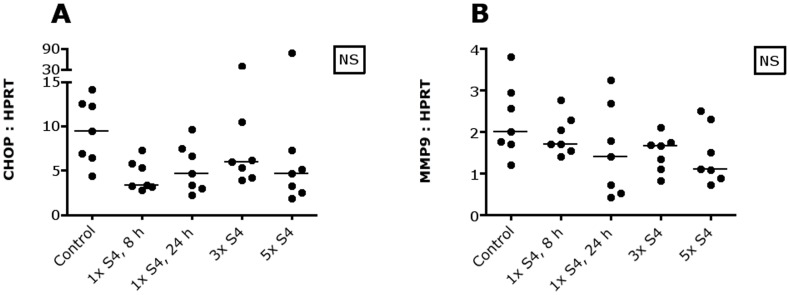
Intracellular pH and CAIX inhibition in SCCNij202. S4 does not change CHOP mRNA (A) and MMP9 mRNA (B). Abbreviations: CHOP, C/EBP homologous protein; MMP9, matrix metallopeptidase 9; NS, not significant; 1× S4, 8 h, one i.p. injection S4, harvest after 8 hours; 1× S4, 24 h, one i.p. injection S4, harvest after 24 hours; 3× S4, one i.p. injection S4 a day for 3 days, harvest 8 hours after the last injection; 5× S4, one i.p. injection S4 a day for 5 days, harvest 8 hours after the last injection.

**Figure 5:**
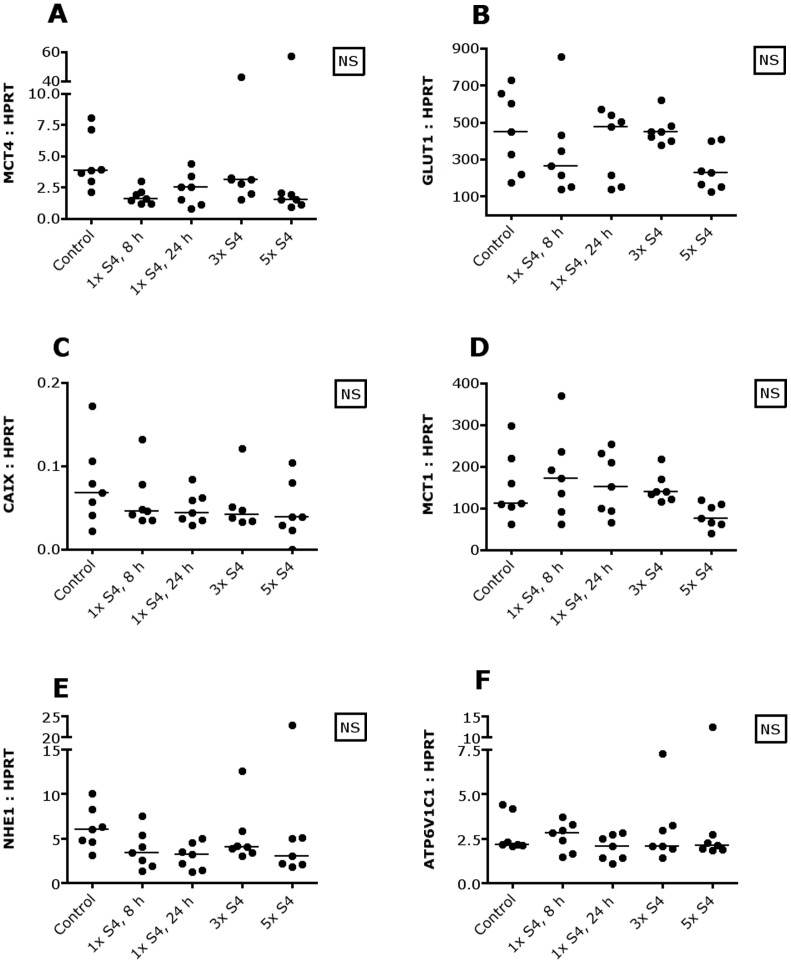
Metabolism and CAIX inhibition in SCCNij202. S4 does not influence the expression of several metabolic transporters and enzymes on the mRNA level. Abbreviations: ATP6V1C1, plasma membrane proton pump vacuolar ATPase (V-ATPase); CAIX, carbonic anhydrase IX; GLUT1, glucose transporter 1; MCT, monocarboxylate transporter; NHE1, sodium-hydrogen exchanger 1; NS, not significant; 1× S4, 8 h, one i.p. injection S4, harvest after 8 hours; 1× S4, 24 h, one i.p. injection S4, harvest after 24 hours; 3× S4, one i.p. injection S4 a day for 3 days, harvest 8 hours after the last injection; 5× S4, one i.p. injection S4 a day for 5 days, harvest 8 hours after the last injection.

To assess whether CAIX inhibition with S4 influences intracellular pH, CHOP mRNA and MMP9 mRNA were measured as CHOP mRNA increases and MMP9 mRNA declines with decreasing intracellular pH [21]. However, no changes in CHOP and MMP9 mRNA were observed (Figure 4). To determine whether tumor cells were able to adapt to CAIX inhibition, thereby counteracting changes in intracellular pH, several transporters and enzymes involved in metabolism and pH regulation were measured. MCT4 mRNA levels changed significantly, but this significance was lost using the Dunn's post test (Figure 5A). Furthermore, no changes in GLUT1, CAIX, MCT1, NHE1 and ATP6V1C1 mRNA were observed (Figure 5B–F).

## Discussion

CAIX has a pivotal role in pH homeostasis and is associated with a poor prognosis in several solid tumors [2], [3]. The sulfamate CAIX inhibitor S4 has been shown to decrease tumor cell proliferation *in vitro* [14], [15]. In this study we examined the effect of S4 on the tumor microenvironment in a laryngeal tumor model.

### CAIX ectodomain shedding

Treatment with S4 decreased CAIX ectodomain shedding, especially 8 hours after the first i.p. injection, indicating that S4 reaches the tumor and that S4 affects the CAIX protein. The ELISA used for the detection of the CAIX ectodomain in serum is specific for human CAIX as both V/10 and M75 antibodies only recognize human and cannot recognize the mouse CAIX [23]. For this reason, it is impossible that the detected CAIX ectodomain in the serum is derived from the mouse. This unequivocally shows that S4 reached the tumor and caused changes in CAIX ectodomain shedding.

The question remains what the biological and clinical meaning of this decrease in shedding is. Enzyme-regulated proteolytic cleavage converts membrane-bound proteins into soluble molecules, a process called ectodomain shedding. In this way, shedding is a regulatory mechanism that influences the expression of proteins on the cell surface and the biological function of these molecules, as these soluble molecules can be biologically active variants. Furthermore, ectodomain shedding can also result in intramembrane protein fragments that may be translocated to the nucleus regulating transcription [26]. CAIX is a very stable protein (half life 38 hours) that undergoes constitutive shedding, which is maintained by moderate hypoxia (2%). However, it is unknown whether the CAIX ectodomain has a function in autocrine or paracrine signaling in cancer biology. Also the potential intracelluar function of intracellular CAIX fragments is under investigation [26].

### CAIX inhibition, the tumor microenvironment and tumor growth

We could not confirm the *in vitro* data that S4 is effective as an antiproliferative agent in this *in vivo* tumor model, as measured by BrdUrd LI [14], [15]. Furthermore, S4 was not pro-apoptotic or pro-necrotic. These observations are fully in line with the data obtained in a HeLa spheroid model, where S4 treatment resulted in a transient decrease in CAIX ectodomain shedding (Pastorekova et al., personal communication). Furthermore, no cytotoxicity as measured by proliferation and cell death was observed, and the effects on spheroid growth were only transient.

Gieling et al. demonstrated that S4 does not affect primary tumor growth in a breast carcinoma *in vivo* model [15]. However, S4 inhibited breast and thyroid cancer cell migration *in vitro* and reduced the number of metastatic tumor colonies in the lung in an orthotopic xenograft tumor model of the human breast cancer line MDA-MB-231 [15]. This implies that S4 could reduce the metastatic burden in the palliative situation and might be able to inhibit metastasis formation when combined with other systemic therapies.

Several preclinical studies have investigated the therapeutic efficacy of other CAIX inhibitors alone and combined with chemo- or radiotherapy [27]–[33]. Treatment with the general CA inhibitor albu-acetazolamide for 3 weeks induced only a partial inhibition of tumor growth in a renal carcinoma xenograft tumor model. Sunitinib, which is used as a first-line treatment for advanced kidney cancer, showed a much stronger delay of tumor growth relative to albu-acetazolamide. Combination therapy was only slightly better compared to treatment with sunitinib alone [27]. Results with albu-acetazolamide were also disappointing in LS174T colorectal cancer xenograft tumors. Treatment with albu-acetazolamide for 2 weeks in this tumor model even led to a slight increase in tumor growth, which however was not statistically significant compared to the control group. Treatment with 5-fluorouracil, which is used in the adjuvant setting for colon carcinoma, yielded a modest tumor growth delay, but the addition of albu-acetazolamide to 5-FU did not offer a therapeutic synergy [27]. So, thus far CAIX inhibitors have not demonstrated more potent anti-tumor activity than current standard first-line treatments.

In contrast to these negative results on primary tumor growth, other preclinical studies examining CAIX inhibition demonstrated more promising results [28], [29]. Dubois et al. studied the therapeutic efficacy of 11c, an indanesulfonamide with high affinity for CAIX, and DH348 in a HT-29 colorectal tumor model [28], [29]. The dual targeting compound DH348 is composed of a 5-nitroimidazole, which mimics oxygen and thereby sensitizes tumors to irradiation, and a CAIX-inhibiting sulfamide. Growth of CAIX-expressing HT-29 tumors was significantly inhibited in mice treated with 11c or DH348 for 5 days versus mice receiving vehicle only, which was further increased upon combination with single dose irradiation. They suggested that the antitumor effect of CAIX inhibition may be linked to intracelluar acidosis resulting in apoptosis and decreased proliferation [29]. The CAIX and CAXII inhibitors ureido-sulfonamide and glycosyl coumarin have been shown to inhibit primary tumor growth and to limit the colonization of metastatic tumor cells in the lung in a 4T1 breast cancer *in vivo* model [30]–[32]. However, the efficacy of CAIX inhibition was not compared with the therapeutic effect of standard first-line treatments in these preclinical studies.

CAIX is expressed in about 95% of clear cell kidney carcinoma. In a clinical phase II trial, 36 patients with progressive metastatic renal cell carcinoma were treated with the CAIX-binding monoclonal antibody WX-G250 (Rencarex) weekly for 12 weeks. Patients with stable disease or response after 12 weeks of treatment continued WX-G250 treatment for additional 8 weeks. Ten patients had stable disease after the first cycle and received extended WX-G250 therapy. Eight of 10 patients still showed stable disease at week 24. One complete response (week 38) and one significant response (week 44) were observed during follow-up. Five patients had stable disease for more than 6 months. The median overall survival was 15 months [34]. The combination of WX-G250 and interferon-alpha-2a in metastatic renal cell carcinoma patients with objective disease progression led to clinically meaningful disease stabilization in 42% of patients, resulting in a significant longer 2-year survival in responders (79%) compared to patients with disease progression (30%) [35]. Thus, a subgroup of patients with kidney carcinoma seems to benefit from this treatment for metastastic disease control.

### CAIX inhibition and pH homeostasis

Several *in vitro* studies have shown the involvement of CAIX in the regulation of cellular pH [5], [28], [29]. Unfortunately, we were not able to demonstrate a decrease in intracellular pH upon treatment with S4, measured by the level of CHOP and MMP9 mRNA as indirect references for intracellular pH [21]. However, CHOP is also strongly induced during hypoxia exposure due to UPR activation, which may confound our findings [36].

Tumors may have compensated for the inhibition of CAIX activity resulting in the lack of effect on proliferation, apoptosis and necrosis. For example, pH regulators might have been not saturated in the untreated situation and thus could have sufficient auxiliary capacity to handle the increase in acids upon treatment with S4. Furthermore, pH regulators can be upregulated through enhanced transcription or activation of proteins. Chiche et al. showed that silencing of CAIX is accompanied by upregulation of CAXII mRNA and protein, resulting in an unchanged extracellular acidification. Furthermore, CAIX was able to compensate for the lack of NHE1, another important pH regulator [7]. In our study, CAIX, MCT1, MCT4, NHE1 and V-ATPase ATP6V1C1 mRNA levels did not change during treatment with S4. Yet, cellular pH homeostasis is a very complex process involving a lot of transporters and enzymes and their isoforms, including monocarboxylate transporters, V-ATPases, sodium-hydrogen exchangers, carbonic anhydrases, chloride/bicarbonate exchangers and natrium/bicarbonate co-transporters amongst others [3], [37]. Compensation might have occurred through one or more isoforms of these regulators at the mRNA or protein level. Another compensatory mechanism could have been a decreased glucose metabolism and thereby a reduced production of acids. GLUT1 mRNA levels did not change in our experiment. However, changes in metabolic activity may have occurred at the protein level, for example through increased degradation or decreased activity of glycolytic transporters and enzymes.

## Conclusions

In this exploratory study, the effect of the sulfamate CAIX inhibitor S4 on the tumor microenvironment was characterized in a laryngeal tumor model. CAIX ectodomain shedding into the blood stream was decreased by S4. As the meaning of this observation is not clear, further studies are required to elucidate whether the CAIX ectodomain has a paracrine or autocrine signalling function in cancer biology. S4 did not influence the tumor microenvironment in a laryngeal tumor model in terms of the amount of proliferation, apoptosis, necrosis and hypoxia. Another preclinical *in vivo* mice study could not demonstrate an effect of S4 on primary tumor growth [15]. Therefore, S4 does not seem to be successful as single agent to influence tumor cell kill (apoptosis, necrosis) and proliferation, and to target primary tumor growth.

## References

[1] Meijer TW, Kaanders JH, Span PN, Bussink J (2012) Targeting hypoxia, HIF-1, and tumor glucose metabolism to improve radiotherapy efficacy. Clin Cancer Res 18: 5585–5594. 23071360 10.1158/1078-0432.CCR-12-0858

[2] Chiche J, Ilc K, Brahimi-Horn MC, Pouyssegur J (2010) Membrane-bound carbonic anhydrases are key pH regulators controlling tumor growth and cell migration. Adv Enzyme Regul 50: 20–33. 19895836 10.1016/j.advenzreg.2009.10.005

[3] Neri D, Supuran CT (2011) Interfering with pH regulation in tumours as a therapeutic strategy. Nat Rev Drug Discov 10: 767–777. 21921921 10.1038/nrd3554

[4] Otero-Rey EM, Somoza-Martin M, Barros-Angueira F, Garcia-Garcia A (2008) Intracellular pH regulation in oral squamous cell carcinoma is mediated by increased V-ATPase activity via over-expression of the ATP6V1C1 gene. Oral Oncol 44: 193–199. 17467328 10.1016/j.oraloncology.2007.02.011

[5] Cianchi F, Vinci MC, Supuran CT, Peruzzi B, De Giuli P, et al (2010) Selective inhibition of carbonic anhydrase IX decreases cell proliferation and induces ceramide-mediated apoptosis in human cancer cells. J Pharmacol Exp Ther 334: 710–719. 20519553 10.1124/jpet.110.167270

[6] Gillies RJ, Robey I, Gatenby RA (2008) Causes and consequences of increased glucose metabolism of cancers. J Nucl Med 49 Suppl 2: 24S–42S. 18523064 10.2967/jnumed.107.047258

[7] Chiche J, Ilc K, Laferriere J, Trottier E, Dayan F, et al (2009) Hypoxia-inducible carbonic anhydrase IX and XII promote tumor cell growth by counteracting acidosis through the regulation of the intracellular pH. Cancer Res 69: 358–368. 19118021 10.1158/0008-5472.CAN-08-2470

[8] Pastorekova S, Zatovicova M, Pastorek J (2008) Cancer-associated carbonic anhydrases and their inhibition. Curr Pharm Des 14: 685–698. 18336315 10.2174/138161208783877893

[9] Supuran CT (2008) Carbonic anhydrases: novel therapeutic applications for inhibitors and activators. Nat Rev Drug Discov 7: 168–181. 18167490 10.1038/nrd2467

[10] Hoogsteen IJ, Marres HA, Wijffels KI, Rijken PF, Peters JP, et al (2005) Colocalization of carbonic anhydrase 9 expression and cell proliferation in human head and neck squamous cell carcinoma. Clin Cancer Res 11: 97–106. 15671533

[11] Ilie MI, Hofman V, Ortholan C, Ammadi RE, Bonnetaud C, et al (2011) Overexpression of carbonic anhydrase XII in tissues from resectable non-small cell lung cancers is a biomarker of good prognosis. Int J Cancer 128: 1614–1623. 20521252 10.1002/ijc.25491

[12] Chien MH, Ying TH, Hsieh YH, Lin CH, Shih CH, et al (2012) Tumor-associated carbonic anhydrase XII is linked to the growth of primary oral squamous cell carcinoma and its poor prognosis. Oral Oncol 48: 417–423. 22172588 10.1016/j.oraloncology.2011.11.015

[13] De Simone G, Alterio V, Supuran CT (2013) Exploiting the hydrophobic and hydrophilic binding sites for designing carbonic anhydrase inhibitors. Expert Opin Drug Discov 8: 793–810. 23627619 10.1517/17460441.2013.795145

[14] Winum JY, Carta F, Ward C, Mullen P, Harrison D, et al (2012) Ureido-substituted sulfamates show potent carbonic anhydrase IX inhibitory and antiproliferative activities against breast cancer cell lines. Bioorg Med Chem Lett 22: 4681–4685. 22721713 10.1016/j.bmcl.2012.05.083

[15] Gieling RG, Babur M, Mamnani L, Burrows N, Telfer BA, et al (2012) Antimetastatic effect of sulfamate carbonic anhydrase IX inhibitors in breast carcinoma xenografts. J Med Chem 55: 5591–5600. 22621623 10.1021/jm300529u

[16] Ljungkvist AS, Bussink J, Kaanders JH, Wiedenmann NE, Vlasman R, et al (2006) Dynamics of hypoxia, proliferation and apoptosis after irradiation in a murine tumor model. Radiat Res 165: 326–336. 16494521 10.1667/rr3515.1

[17] Troost EG, Bussink J, Kaanders JH, van Eerd J, Peters JP, et al (2005) Comparison of different methods of CAIX quantification in relation to hypoxia in three human head and neck tumor lines. Radiother Oncol 76: 194–199. 16024110 10.1016/j.radonc.2005.06.031

[18] van Laarhoven HW, Bussink J, Lok J, Punt CJ, Heerschap A, et al (2004) Effects of nicotinamide and carbogen in different murine colon carcinomas: immunohistochemical analysis of vascular architecture and microenvironmental parameters. Int J Radiat Oncol Biol Phys 60: 310–321. 15337570 10.1016/j.ijrobp.2004.05.014

[19] Stegeman H, Kaanders JH, Wheeler DL, van der Kogel AJ, Verheijen MM, et al (2012) Activation of AKT by hypoxia: a potential target for hypoxic tumors of the head and neck. BMC Cancer 12: 463. 23046567 10.1186/1471-2407-12-463 PMC3517352

[20] Stegeman H, Kaanders JH, van der Kogel AJ, Iida M, Wheeler DL, et al (2013) Predictive value of hypoxia, proliferation and tyrosine kinase receptors for EGFR-inhibition and radiotherapy sensitivity in head and neck cancer models. Radiother Oncol 106: 383–389. 23453541 10.1016/j.radonc.2013.02.001 PMC3627829

[21] Putney LK, Barber DL (2004) Expression profile of genes regulated by activity of the Na-H exchanger NHE1. BMC Genomics 5: 46. 15257760 10.1186/1471-2164-5-46 PMC499544

[22] Zatovicova M, Sedlakova O, Svastova E, Ohradanova A, Ciampor F, et al (2005) Ectodomain shedding of the hypoxia-induced carbonic anhydrase IX is a metalloprotease-dependent process regulated by TACE/ADAM17. Br J Cancer 93: 1267–1276. 16278664 10.1038/sj.bjc.6602861 PMC2361518

[23] Zat'ovicova M, Tarabkova K, Svastova E, Gibadulinova A, Mucha V, et al (2003) Monoclonal antibodies generated in carbonic anhydrase IX-deficient mice recognize different domains of tumour-associated hypoxia-induced carbonic anhydrase IX. J Immunol Methods 282: 117–134. 14604546 10.1016/j.jim.2003.08.011

[24] Westbury CB, Pearson A, Nerurkar A, Reis-Filho JS, Steele D, et al (2007) Hypoxia can be detected in irradiated normal human tissue: a study using the hypoxic marker pimonidazole hydrochloride. Br J Radiol 80: 934–938. 17908818 10.1259/bjr/25046649

[25] McDonald PC, Winum JY, Supuran CT, Dedhar S (2012) Recent developments in targeting carbonic anhydrase IX for cancer therapeutics. Oncotarget 3: 84–97. 22289741 10.18632/oncotarget.422 PMC3292895

[26] Zatovicova M, Pastorekova S (2013) Modulation of cell surface density of carbonic anhydrase IX by shedding of the ectodomain and endocytosis. Acta Virol 57: 257–264. 23600882 10.4149/av_2013_02_257

[27] Ahlskog JK, Dumelin CE, Trussel S, Marlind J, Neri D (2009) In vivo targeting of tumor-associated carbonic anhydrases using acetazolamide derivatives. Bioorg Med Chem Lett 19: 4851–4856. 19615903 10.1016/j.bmcl.2009.06.022

[28] Dubois L, Peeters S, Lieuwes NG, Geusens N, Thiry A, et al (2011) Specific inhibition of carbonic anhydrase IX activity enhances the in vivo therapeutic effect of tumor irradiation. Radiother Oncol 99: 424–431. 21676479 10.1016/j.radonc.2011.05.045

[29] Dubois L, Peeters SG, van Kuijk SJ, Yaromina A, Lieuwes NG, et al (2013) Targeting carbonic anhydrase IX by nitroimidazole based sulfamides enhances the therapeutic effect of tumor irradiation: A new concept of dual targeting drugs. Radiother Oncol 10.1016/j.radonc.2013.06.018 23849171

[30] Lou Y, McDonald PC, Oloumi A, Chia S, Ostlund C, et al (2011) Targeting tumor hypoxia: suppression of breast tumor growth and metastasis by novel carbonic anhydrase IX inhibitors. Cancer Res 71: 3364–3376. 21415165 10.1158/0008-5472.CAN-10-4261

[31] Pacchiano F, Carta F, McDonald PC, Lou Y, Vullo D, et al (2011) Ureido-substituted benzenesulfonamides potently inhibit carbonic anhydrase IX and show antimetastatic activity in a model of breast cancer metastasis. J Med Chem 54: 1896–1902. 21361354 10.1021/jm101541x

[32] Touisni N, Maresca A, McDonald PC, Lou Y, Scozzafava A, et al (2011) Glycosyl coumarin carbonic anhydrase IX and XII inhibitors strongly attenuate the growth of primary breast tumors. J Med Chem 54: 8271–8277. 22077347 10.1021/jm200983e

[33] Gieling RG, Parker CA, De Costa LA, Robertson N, Harris AL, et al (2013) Inhibition of carbonic anhydrase activity modifies the toxicity of doxorubicin and melphalan in tumour cells in vitro. J Enzyme Inhib Med Chem 28: 360–369. 23163664 10.3109/14756366.2012.736979

[34] Bleumer I, Knuth A, Oosterwijk E, Hofmann R, Varga Z, et al (2004) A phase II trial of chimeric monoclonal antibody G250 for advanced renal cell carcinoma patients. Br J Cancer 90: 985–990. 14997194 10.1038/sj.bjc.6601617 PMC2410216

[35] Siebels M, Rohrmann K, Oberneder R, Stahler M, Haseke N, et al (2011) A clinical phase I/II trial with the monoclonal antibody cG250 (RENCAREX(R)) and interferon-alpha-2a in metastatic renal cell carcinoma patients. World J Urol 29: 121–126. 20512580 10.1007/s00345-010-0570-2

[36] Rouschop KM, van den Beucken T, Dubois L, Niessen H, Bussink J, et al (2010) The unfolded protein response protects human tumor cells during hypoxia through regulation of the autophagy genes MAP1LC3B and ATG5. J Clin Invest 120: 127–141. 20038797 10.1172/JCI40027 PMC2798689

[37] Xu K, Mao X, Mehta M, Cui J, Zhang C, et al (2013) Elucidation of How Cancer Cells Avoid Acidosis through Comparative Transcriptomic Data Analysis. PLoS One 8: e71177. 23967163 10.1371/journal.pone.0071177 PMC3743895

